# Three-Step Enzymatic Remodeling of Chitin into Bioactive Chitooligomers

**DOI:** 10.1021/acs.jafc.4c03077

**Published:** 2024-07-09

**Authors:** Zuzana Mészáros, Natalia Kulik, Lucie Petrásková, Pavla Bojarová, Mònica Texidó, Antoni Planas, Vladimír Křen, Kristýna Slámová

**Affiliations:** † Institute of Microbiology of the Czech Academy of Sciences, Vídeňská 1083, CZ 14200, Prague 4, Czech Republic; ‡ Laboratory of Biochemistry, Institut Químic de Sarrià, University Ramon Llull, ES 08017 Barcelona, Spain

**Keywords:** chitin, chitinase, chitooligomer, β-*N*-acetylhexosaminidase, peptidoglycan deacetylase

## Abstract

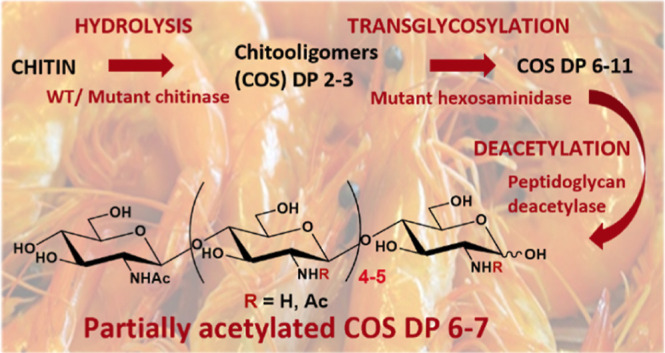

Here we describe a complex enzymatic approach to the efficient transformation of abundant waste chitin, a byproduct of the food industry, into valuable chitooligomers with a degree of polymerization (DP) ranging from 6 to 11. This method involves a three-step process: initial hydrolysis of chitin using engineered variants of a novel fungal chitinase from *Talaromyces flavus* to generate low-DP chitooligomers, followed by an extension to the desired DP using the high-yielding Y445N variant of β-*N*-acetylhexosaminidase from *Aspergillus oryzae*, achieving yields of up to 57%. Subsequently, enzymatic deacetylation of chitooligomers with DP 6 and 7 was accomplished using peptidoglycan deacetylase from *Bacillus subtilisBs*PdaC. The innovative enzymatic procedure demonstrates a sustainable and feasible route for converting waste chitin into unavailable bioactive chitooligomers potentially applicable as natural pesticides in ecological and sustainable agriculture.

## Introduction

Chitooligomers (COS, β-1,4-linked oligomers of *N*-acetylglucosamine (GlcNAc) and glucosamine (GlcN)) are bioactive compounds with diverse beneficial effects, featuring mainly antitumor, immunomodulatory, antiangiogenic, antioxidant, and antimicrobial properties.^1^ They activate plant defense mechanisms and indirectly protect plants from diseases as elicitors of resistance against bacterial, fungal, and insect pathogens.^2,3^ Chitin, the source of COS, is abundant in crustacean shells, creating a considerable amount of biowaste (6–8 million tons/year) in the seafood industry.^4^ The biological activity of COS depends on factors like the degree of polymerization (DP) and the degree of *N*-acetylation (DA). Longer COS of DP 6–8 exhibit higher antifungal activity and stronger affinity to the respective receptors than shorter COS of DP 4–5, thus inducing chitin-responsive genes more effectively.^5,6^ However, fully *N*-acetylated COS with DP ≥ 7 have low solubility, which limits their bioavailability. Partial deacetylation enhances solubility and biological activity of higher-DP COS.^7^

Chitooligomers have been traditionally obtained by hydrolysis of chitin either chemically using concentrated acids or enzymatically by microbial chitinases. Typically, these methods generate complex mixtures of low-DP COS. Moreover, chemical hydrolysis of chitin requires harsh conditions using concentrated acids at high temperatures, affording highly salted COS products upon neutralization of the hydrolytic reaction. On the other hand, chitinases can be employed for chitin hydrolysis under mild aqueous conditions, however, they suffer from lower hydrolytic efficiency as crystalline chitin is not an easily accessible substrate.^1^ Enzymatic synthesis of COS with higher DP faces challenges in converting chitin to well-defined chitooligomers. The past decade saw a rapid development of enzymatic synthetic methods for oligosaccharides using engineered glycosidases.^8^ However, a robust method for the green conversion of the underexploited chitin waste to highly valuable and well-defined chitooligomers of DP ≥ 6—fully and/or partially acetylated—remains a challenge. Scalable production of COS with defined DP and DA is crucial for the structure–activity relationship studies, mainly concerning the COS effects on eliciting plant defense mechanisms against various pathogens. It is an important up-to-date issue of sustainable agriculture, which can reduce the consumption of harmful pesticides in crop protection.^9^

Chitinases (GH18; EC 3.2.1.14) and β-*N*-acetylhexosaminidases (GH20; EC 3.2.1.52) are enzymes crucial for the degradation of chitin both in growing fungi, insects, and crustaceans, and for nutrition.^8^ Both enzyme families use the so-called substrate-assisted catalytic mechanism, in which the substrate 2-acetamido moiety acts as an intramolecular nucleophile instead of an enzyme residue, and an oxazoline reaction intermediate is formed.^10,11^ In our previous work, we focused on the engineering of fungal β-*N*-acetylhexosaminidases, achieving hypertransglycosylating synthetic engines with outstanding capabilities to synthesize derivatized chitooligomers.^12,13^ The first hypertransglycosylating mutants of fungal β-*N*-acetylhexosaminidases were prepared by the mutagenesis of the oxazoline-stabilizing tyrosine residue^14^ and of the assisting aspartate residue.^15^ Moreover, superior GH20 transglycosidases prepared by the combined mutagenesis of the tyrosine residue and the aglycone-binding aromatic residues were recently reported.^13^ On the other hand, GH18 chitinases are the enzymes of choice when the hydrolysis of chitin is concerned.^16^ The enzymatic hydrolysis of chitin is challenging as the substrate is insoluble and highly recalcitrant with a rigid crystalline structure. Therefore, novel engineered chitinases with increased hydrolytic activities are of eminent importance and attractiveness for chitin processing.^17,18^

Enzymatic deacetylation of chitooligosaccharides is performed by chitin deacetylases. These enzymes, together with peptidoglycan deacetylases, belong to the carbohydrate esterase CE4 family of Carbohydrate Active Enzymes (www.cazy.org).^19^ CE4 enzymes catalyze de-*N*-acetylation of chitin or peptidoglycan by a metal-assisted general acid/base catalysis. Some peptidoglycan deacetylases are also active on chitooligomers, with potential application in partial deacetylation of the chitin degradation products from chitinases and β-*N*-acetylhexosaminidases. The peptidoglycan deacetylase from *Bacillus subtilisBs*PdaC was previously identified as the enzyme with dual specificity due to its additional capacity to deacetylate chitooligomers besides peptidoglycan,^20,21^ therefore, it was used in this study.

**Figure 1:**
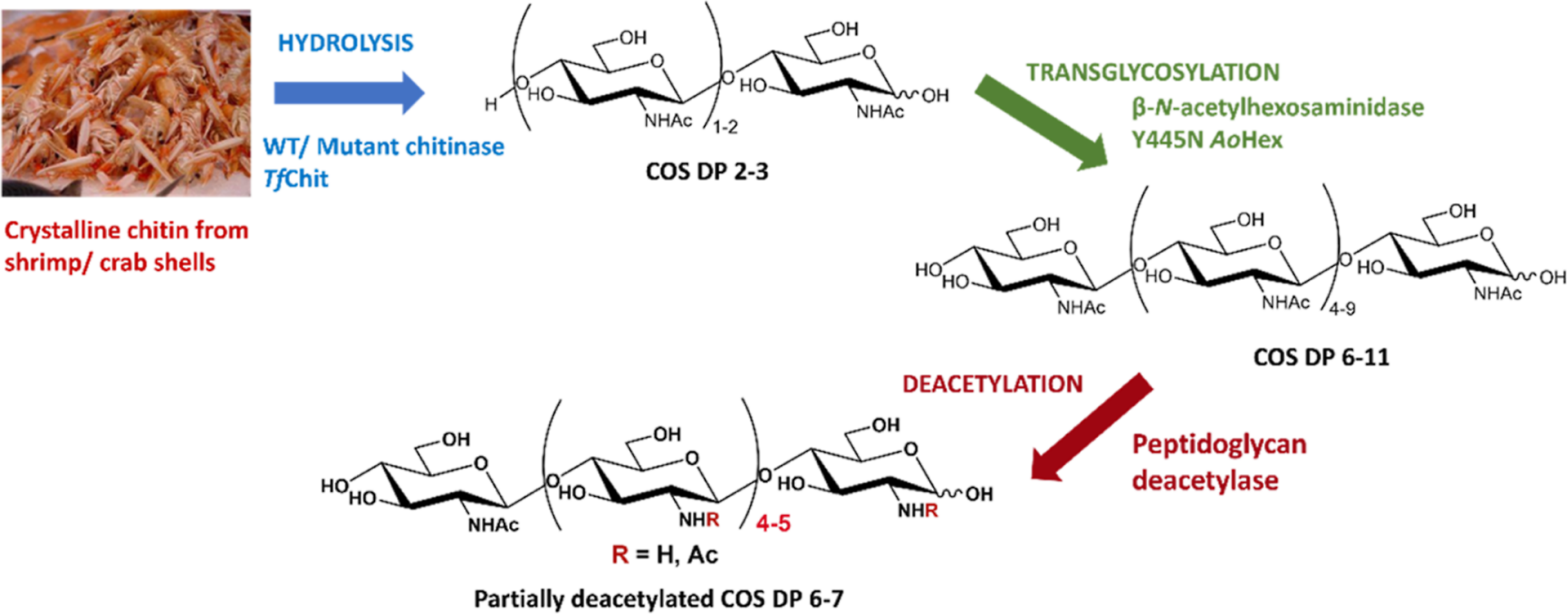
Schematic presentation of the three-step process for the enzymatic transformation of chitin into partially deacetylated chitooligomers.

This work presents a sequential three-step enzymatic route for the green and sustainable treatment of chitin biowaste, leading to the efficient synthesis of COS with desired DP ≥ 6 (Figure 1). Engineered variants of the chitinase from *Talaromyces flavus* (*Tf*Chit) were employed for effective hydrolysis of chitin and high-yielding preparation of low-DP COS. Subsequently, the mutant variant of β-*N*-acetylhexosaminidase from *Aspergillus oryzae* (*Ao*Hex) was employed in the high-yielding synthesis of COS with DP 6–11. In the last step, the peptidoglycan deacetylase from *B. subtilis* (*Bs*PdaC) was used in the pilot experiment of enzymatic deacetylation of the mixture of insoluble chitooligomers.

## Materials and Methods

Unless otherwise stated, the chemicals used in this study were of analytical grade from Sigma-Aldrich (CZ) or VWR Chemicals (CZ).

### Expression and Characterization of *Tf*Chit

The gene encoding for chitinase from *T. flavus* (*Tf*Chit; GenBank ID: ADV02753.1) was prepared synthetically on a commercial basis with codon usage optimized for its expression in *Pichia pastoris* (Generay Biotech, CN) and cloned into the yeast expression vector pPICZαA via the 5′-*Eco*RI and 3′-*Kpn*I restriction sites forming the expression construct pPICZαA-*Tf*Chit. The prepared construct was used for the extracellular expression of *Tf*Chit enzyme in *P. pastoris* KM71H (Invitrogen, US) as previously described for the fungal β-*N*-acetylhexosaminidase *Ao*Hex in detail.^13^

For the preparatory production of *Tf*Chit, BMGH medium (Buffered Minimal Glycerol medium) and BMMH medium (Buffered Minimal Methanol medium) were used, starting with a preculture; the expression of *Tf*Chit was induced by the addition of methanol (0.5% *v*/*v*) every 24 h. The produced chitinase was purified from the culture medium after 3 days of cultivation by centrifugation at 5000*g* for 10 min. The medium was diluted 3× with water and its pH was adjusted to 6.5. The final medium was filtered and loaded on the Q-Sepharose column (GE Healthcare, SW) pre-equilibrated with 10 mM sodium citrate-phosphate buffer pH 6.5, and the enzyme was eluted using a linear gradient of NaCl (0–1 M) on an Äkta Purifier LC system. The purity of the fractions was checked by 10% SDS-PAGE and protein concentration was determined by the Bradford assay using Protein Assay Dye Reagent Concentrate and bovine γ-globulin as a protein standard (Bio-Rad, US). Fractions containing *Tf*Chit were pooled and concentrated; the buffer was exchanged for 50 mM sodium citrate-phosphate pH 5.0 and the purified enzyme was stored at 4 °C for several months without any loss of activity.

### Preparation of *Tf*Chit Mutant Variants

For the site-directed mutagenesis of *Tf*Chit, the expression construct pPICZαA-*Tf*Chit was used as a template. The PCR reactions were performed in a T-Personal Thermal Cycler (Biometra, DE) using QuikChange Lightning Site-Directed Mutagenesis Kit (Agilent Technologies, US), the sequences of the primers for the PCR reactions are given in the Supporting Information (Tables S1 and S2). After PCR, 1 μL of *Dpn*I (New England Biolabs, US) was added to the reaction mixture to cleave the template plasmid DNA, and the reaction was incubated for 2 h at 37 °C. The PCR product was isolated from the agarose gel using JETQUICK Gel Extraction Spin Kit (Genomed, DE), transformed into the *E. coli* TOP10 (Promega, US) strain, and the cells were spread on LB agar plates with zeocin (Invitrogen, US) as a selection marker. The plasmid was isolated from the colonies cultured in the LB media overnight (37 °C) using Genopure Plasmid Midi Kit (Roche, US), and the sequences of the mutant genes were confirmed by Sanger sequencing (Seqme, CZ). The mutant variants of *Tf*Chit were expressed and purified as described above.

### Production and Purification of Y445N *Ao*Hex

The Y445N variant of *Ao*Hex was prepared, expressed in *P. pastoris*, and purified using cation-exchange chromatography as described previously.^13^

### Chitinase and β-*N*-Acetylhexosaminidase Activity and Kinetic Assays

The catalytic activity was measured spectrophotometrically using *p*NP-GlcNAc for β-*N*-acetylhexosaminidases and *p*NP-CH3 (*p*-nitrophenyl *N*,*N*′,*N*″-triacetyl-β-chitotrioside) for chitinases at 2 mM starting concentration and 10 μL of enzyme. The concentrations for each enzyme variant was slightly different, however, it was in the range of nanomolar concentrations of the enzymes or lower (10^–6^–10^–7^ mol/L). The reaction mixture (50 μL) was incubated in 50 mM citrate/phosphate buffer pH 5.0 for 10 min at 35 °C and 1000 rpm (Thermomixer Comfort, Eppendorf, DE). Then, the reaction was stopped by adding 0.1 M Na_2_CO_3_ (1 mL), and the concentration of released *p*-nitrophenol was determined spectrophotometrically at 420 nm. One unit (U) of enzymatic activity was defined as the amount of enzyme releasing 1 μmol of *p*-nitrophenol per minute. Michaelis–Menten kinetics was measured in an end-point assay described above at 420 nm using *p*NP-CH3 as substrate in the concentration range of 0.1–6 mM at 25 °C. GraphPad Prism was used to calculate the kinetic parameters. All data were measured in three parallel experiments.

### Hydrolysis of Chitin by *Tf*Chit and its Variants

The reaction mixture contained chitin (50 mg; crystalline chitin from crustacean shells, Biosynth, UK) as a substrate, 25 mM citrate-phosphate buffer pH 5.0, and 1 mg of *Tf*Chit or its variant (total reaction volume 5 mL). The enzyme was dialyzed overnight against 25 mM citrate-phosphate buffer pH 5.0 to decrease and standardize the amount of salts in the reaction mixture. The reaction was incubated at 35 °C with shaking (1000 rpm). After 48 h, the reaction was stopped by boiling (99 °C, 5 min) and the reaction mixtures were centrifuged. The supernatant was lyophilized and weighed. The blank reaction was void of enzyme.

### Molecular Modeling of *Tf*Chit

BLAST search^22^ was used to identify homologues of *Tf*Chit (ADV02753.1) in the Protein Databank (accessed in December 2021).^23^ Multiple sequence alignment was built with Clustal Omega^24^ and modified in Jalview.^25^ The secondary structure assignment was done with ESPript.^26^ From the identified homologues of *Tf*Chit the structures of chitinases from *Aspergillus fumigatus* (PDB code 2a3e)^27^ and *Aspergillus niger* (6igy)^28^ were used for the construction of a homology model in complex with CH6 (*N*,*N*′,*N*″,*N*‴,*N*⁗,*N*′′′′′-hexaacetylchitohexaose; chitohexaose) with Modeler 9.20.^29^ Additionally, the structure of Chitinase from *Ostrinia furnacalis* (5wvb)^30^ with a lower identity of 30.15% was used for the proper placement of the loop 348–358 and the addition of CH6. YASARA standard protocol^31^ was used to refine the homology model in the 50 ns molecular dynamics (MD) simulation. During equilibration, the active site residues defined as residues within 0.3 nm from the docked hexasaccharide were fixed. Structure quality and stability were analyzed with Vadar^32^ and YASARA.

MD simulations were run with YASARA using a Glycam force field for carbohydrates,^33^ general amber force field parameters for modified parts of ligands, and a YASARA2 force field for protein and solvent (water and ions) at the *NPT* ensemble.^34^ Structure alignments were performed in YASARA employing the MUSTANG algorithm.^35^ Protonation states of the catalytic residues were set based on the proposed catalytic mechanism of GH18 chitinases^36^ with YASARA. Residues were mutated with YASARA, followed by short energy minimization using the standard protocol.^31^ Modification of ligands was done based on the cocrystallized hexasaccharide in the active site of chitinase from *O. furnacalis*,^30^ and the ligand position was refined by energy minimization in YASARA.^31^

The analysis of water-mediated interactions was done by the Watclust program.^37^ The positions including water present in more than 20% of MD simulations were accepted as occupied. The binding score after MD was calculated with Vina (score in place) and averaged over 3 selected snapshots.^38^ All averaged data were calculated from 10 ns of equilibrated period of MD simulations (40–50 ns). The main reason for the selection of this period was that by this time ligand RMSD was stable and its interaction with catalytic Glu138 was preserved. For longer simulations, this catalytic residue lost interaction with ligands even in the WT *Tf*Chit.

### Transglycosylation Reactions with Engineered *Ao*Hex for the Synthesis of Insoluble COS

The reaction mixtures contained 50 mM citrate-phosphate buffer pH 5.0, CH3 (*N*,*N*′,*N*″-triacetylchitotriose; chitotriose; 100 mM) as a substrate, 0.25 U of enzyme *Ao*Hex Y445N or *Ao*Hex V306W/Y445F;^13^ the total reaction volume was 480 μL. The reactions were incubated at 35 °C under vigorous shaking (1000 rpm). After 24 h, the reaction mixture was stopped by boiling (5 min), cooled, and centrifuged, and the pellet of insoluble COS products was collected. Additional substrate (CH3, 15 mg) was added to the supernatant and incubation was continued. After 24 h, the reaction was stopped by boiling and then centrifuged. The sediments (products) were subsequently pooled, lyophilized, and weighed. In the analytical reactions with COS of various DP, 0.25 or 0.5 U of *Ao*Hex Y445N and 100 mM of CH2 (*N*,*N*′-diacetylchitobiose; chitobiose), CH3, and CH4 (*N*,*N*′,*N*″,*N*‴-tetraacetylchitotetraose; chitotetraose) were incubated in a microtiter plate (pH 5, 35 °C, total volume 0.2 mL) with shaking (300 rpm). In defined time intervals the optical density of the reaction mixture was measured spectrophotometrically at 600 nm. In the semipreparatory transglycosylation reactions, 30 mg of CH2, CH3, or CH4 (100 mM) were incubated with 0.25–1 U of *Ao*Hex Y445N (pH 5, 35 °C, 1000 rpm) for 24 h. Then the reaction was stopped by boiling for 5 min, cooled, and centrifuged and the pellet of the insoluble COS was collected, lyophilized, and weighed. The mixture of insoluble COS products was analyzed by MALDI-TOF MS (Figure S11).

### Production and Purification of Peptidoglycan Deacetylase *Bs*PdaC

*Bs*PdaC was expressed and purified as reported.^20^ A colony of transformed *E. coli* BL21(DE3) cells containing plasmid pET22b_PdaC-CD was grown in 5 mL of LB medium containing ampicillin (100 μg/mL) for 7 h at 37 °C and 250 rpm. This preculture was then inoculated into 0.5 L of autoinduction medium with ampicillin (100 μg/mL) in a 2 L Erlenmeyer flask and incubated for 48 h at 25 °C and 170 rpm. The culture was then centrifuged, and the pellet was suspended in 30 mL of PBS buffer (50 mM Na_2_HPO_4_, 300 mM NaCl) pH 7.5 containing 1 mM serine protease inhibitor phenylmethylsulfonyl fluoride (PMSF) and disrupted by sonication using a Vibra Cell VCX 130 sonicator (Sonics, US) with a 6 mm probe (7 min, 10 s on and 15 s off, 50% amplitude). The cell-free extract was centrifuged (12,000 rpm, 1 h at 4 °C) with Avanti J-26 XPI High-Performance Centrifuge (Beckman Coulter, US). The supernatant was filtered through a 0.45 μm filter. After the supernatant was collected, two chromatographic steps employing the ÄKTA FPLC system (Amersham Biosciences, US) were performed to isolate the enzyme from the cell lysate: affinity chromatography using a 1 mL StrepTrap column (Sigma-Aldrich, DE), followed by size exclusion chromatography using a Superdex 200 (16/600) column (GE Healthcare, US). The fractions containing *Bs*PdaC were pooled, concentrated by ultrafiltration using Amicon Ultra-15 Centrifugal Filter Devices (MWCO 10 kDa) (Merck Millipore, DE), and stored at 4 °C.

### Deacetylation of Chitooligomers by *Bs*PdaC

Enzymatic deacetylation reactions were performed with COS of DP4, DP5, and a mixture of insoluble COS (from the previous transglycosylation reactions, DP 6 to 11) as substrates in PBS buffer (50 mM Na_2_HPO_4_, 300 mM NaCl) at pH 7.5 and 37 °C. The formation of products of different degrees of deacetylation was monitored by HPLC-MS (Agilent 1260 HPLC-MS, electrospray ionization (ESI+), single quadrupole MS detector) using an XBridge BEH Amide 2.5 μM, 3.0 × 100 mm XP column (Waters, NL) in combination with an XBridge BEH Amide Guard Cartridge (2PK) precolumn (2.5 μM × 20 mm). Samples (5 μL) were injected into the system and eluted at 60 °C with acetonitrile/water (65:35, *v*/*v*)/1% formic acid at a flow rate of 0.4 mL/min. MS detection was performed on both SIM mode (for monitoring [M + H]^+^ of substrate and deacetylation products, Table S4) and SCAN mode (for total ion monitoring, 250–1100 *m*/*z* scan range).

(a) Enzyme activity with reference DP4 substrate (CH4). 80 μL of the reaction mixture (2.5 mM CH4 in PBS buffer pH 7.5) were placed in a microtiter plate well, whereas the enzyme solution (22 μL at different *Bs*PdaC concentrations for each reaction) was placed in another well of the same plate. The plate was incubated at 37 °C for 10 min. To start the reaction, 20 μL of the enzyme was added to 80 μL of the reaction mixture. The final reaction conditions were: 2 mM substrate, 1 to 10 μM enzyme in 50 mM Na_2_HPO_4_, 300 mM NaCl at pH 7.5 and 37 °C. Then, aliquots of 10 μL were collected at regular intervals (1, 2, 5, 10, 15, 20, 30, and 45 min) and mixed with 90 μL of STOP solution (1-propanol/H_2_O 1:1, v/v) that were located in the STOP microplate. Samples (5 μL) were analyzed by HPLC-MS as described above. These experiments allowed us to select the appropriate enzyme concentrations to perform the enzymatic deacetylation of insoluble chitooligomers.
(b) Deacetylation of chitooligomers. Four reactions were run to monitor the deacetylation time course: A and B: 2 mM substrate (CH4 and CH5, respectively) and 4 μM *Bs*PdaC enzyme in PBS pH 7.5; C: 10 mg of insoluble COS (DP 6–11) and 20 μM *Bs*PdaC enzyme in 10 mM NH_4_HCO_3_ pH 7.5; D: 10 mg of insoluble COS (DP 6–11) and 20 μM *Bs*PdaC enzyme in 0.5% Triton X-100 in water. The reaction mixtures were incubated for 120 h at 37 °C and 300 rpm. At 24, 48, and 120 h, 30 μL aliquots were collected and added to 270 μL of the STOP solution (1-propanol/H_2_O 1:1, v/v). Then, 5 μL samples were analyzed by HPLC-MS as described above. The time course monitoring of each reaction is presented in Figure 6.

## Results and Discussion

### Preparation and Characterization of *Tf*Chit and its Mutant Variants

As our research is focused on the discovery and characterization of new enzymes with biotechnologically interesting activities and properties, a new fungal chitinase from *T. flavus* (*Tf*Chit) was selected for study and engineering in order to increase its hydrolytic activity with chitin substrate. The selected chitinase sequence shares 63% identity with another fungal chitinase from *A. fumigatus*, which is relatively modest and suggests the potential for functional and structural divergences. *Tf*Chit and its mutant variants were extracellularly produced in the yeast expression system of *P. pastoris* and purified in one step by anion-exchange chromatography from its culture media. Nine mutant variants of *Tf*Chit with proposed increased hydrolytic activity were designed by molecular modeling, targeting the −1 subsite (catalytic residue D136) and the +2/+3 subsites; W209 was mutated to Ala, and S181 and F231 were changed for tryptophan to enhance the interactions of the enzyme with the substrate. Substitution residues were checked for clashes with the substrate. The mutation of the catalytic amino acid D136 was proposed based on the experience with the mutation in the related β-*N*-acetylhexosaminidase from *T. flavus*, where the replacement of the catalytic Asp with Val significantly increased its hydrolytic activity.^15^ The W209A, F231W, and S181W were based on literature studying the effects of the presence of Trp residues in the aglycone binding sites (+1 to +3), which influence the binding of the longer substrates to the active site of chitinases.^39^ Subsequently, the mutations were also combined, resulting in four double mutants (W209A/D136V, W209A/F231W, S181W/D136V, S181W/W209A) and one triple mutant (S181W/W209A/D136V).

Interestingly, most of the mutant variants of *Tf*Chit had a higher specific activity than the parent enzyme with the *p*NP-CH3 substrate (Table 1). A significant increase in *K*_M_ was observed in the W209A, W209A/D136V, and S181W/D136V mutants, ca. 3× compared to WT *Tf*Chit, reflecting their lower affinity to the substrate. However, all mutants (except for F231W and S181W/W209A) exhibited an increased *k*_cat_ parameter. Altogether, the resulting specificity constant *k*_cat_/*K*_M_ increased only in two variants (D136V, S181W), and the values of this ratio stayed in a very narrow range from 1.2 to 5.0 L·s^–1^·mmol^–1^ (Table 1). Thus, the kinetic data obtained with the chromogenic substrate *p*NP-CH3 showed no substantial differences in the activity of the prepared *Tf*Chit variants, however, significant variation in the hydrolysis of the natural substrate chitin was observed as described below.

**Table 1:** Kinetic Parameters of the Hydrolysis of the Chromogenic Substrate *p*NP-CH3 by Chitinase from *Talaromyces flavus* (*Tf*Chit) and its Variants

*Tf*Chit variant	specific activity [U·mg^–1^]	*K*_M_ [mmol·L^–1^]	*k*_cat_ [s^–1^]	*k*_cat_/*K*_M_ [L·s^–1^·mmol^–1^]
WT	2.1	1.2	3.9	3.3
D136V	2.1	1.1	5.3	5.0
W209A	3.1	3.3	6.1	1.9
S181W	4.8	1.5	6.0	4.0
F231W	1.2	1.1	1.9	1.8
W209A/D136V	6.2	3.6	8.2	2.2
W209A/F231W	4.7	1.3	4.7	3.5
S181W/D136V	6.5	4.3	5.3	1.2
S181W/W209A	4.7	2.6	3.1	1.2
S181W/W209A/D136V	3.9	2.0	5.6	2.8

### Hydrolysis of Chitin by *Tf*Chit and its Mutant Variants

Enzyme engineering of *Tf*Chit was aimed at increasing its hydrolytic activity to promote the hydrolysis of the recalcitrant polysaccharide substrate chitin. Therefore, the preparatory reactions were performed to determine the capabilities of the individual *Tf*Chit variants to hydrolyze chitin to low-DP chitooligomers. Table 2 presents the results of chitin hydrolysis using WT *Tf*Chit and its engineered variants with the proposed increased hydrolytic activity, which was determined as the amount of soluble COS obtained from 50 mg of chitin after 48 h reaction. Of the nine *Tf*Chit variants prepared, only one was inferior in the hydrolytic ability compared to WT *Tf*Chit. The best results were achieved with single mutants W209A and F231W *Tf*Chit, for which the yield of chitin hydrolysis was more than doubled compared to WT *Tf*Chit. The third most efficient enzyme was the triple mutant, which produced almost double the amount of COS than the parent enzyme. Surprisingly, the double mutants performed worse than the enzymes with single mutations and the increase in chitin hydrolysis was less pronounced. As mentioned above, the differences in the hydrolysis of the natural substrate chitin by the *Tf*Chit variants were much more profound than observed in the enzyme kinetics with the artificial substrate *p*NP-CH3 (Tables 1 and 2). Interestingly, the *Tf*Chit variants with most increased specificity constant (D136V, S181W, W209A/F231W) are different from the variants that performed best with chitin (W209A, F231W, triple mutant). We can conclude that there are other interactions that were not identified by molecular modeling and enzyme kinetics that significantly affect the processing of the crystalline substrate by the enzymes.

**Table 2:** Chitin Hydrolysis Using WT Chitinase from *Talaromyces flavus* (*Tf*Chit) and its Mutant Variants (50 mg Chitin, pH 5.0, 1 mg Enzyme, 35 °C, 48 h)^a^

*Tf*Chit variant	hydrolytic products [mg]	chitin hydrolysis rate [mg·h^–1^·mgE^–1^]	hydrolysis related to WT [%]
WT	9.5	0.20	100
D136V	13.7	0.29	144
W209A	19.9	**0.41**	**209**
S181W	10	0.21	105
F231W	21.1	**0.44**	**222**
W209A/D136V	9.7	0.20	102
W209A/F231W	14.3	0.30	151
S181W/D136V	7	0.15	74
S181W/W209A	11.5	0.24	121
S181W/W209A/D136V	17.5	**0.36**	**184**

a The best results are highlighted in bold. The chitin hydrolysis rate is defined as the mass of the soluble chitooligomers formed in 1 h per 1 mg of enzyme.

Overall, it is very difficult to compare the effectivity of *Tf*Chit with other chitinases reported in the literature as there are various substrates, conditions, reaction times, and analytical methods used. Moreover, there is only scarce information on the successful engineering of chitinases to increase their hydrolytic activity toward chitin. As an example, Visootsat and co-workers obtained double and triple mutant variants of chitinase A from *Serratia marcescens* able to hydrolyze crystalline chitin with increased efficiency.^17^ In all *Tf*Chit variants, the major products of chitin hydrolysis were disaccharide CH2 and trisaccharide CH3, with the former slightly predominant, as determined by HPLC analysis (Figure S1). The predominant formation of chitobiose as a product of chitin hydrolysis is typically found in processive chitinases,^25,40^ which is probably the case of *Tf*Chit.

### Molecular Modeling of *Tf*Chit

Enzyme–substrate complexes were modeled to rationalize the structural effects of the tested mutations on substrate hydrolysis. The homology model of the chitinase from *T. flavus* (*Tf*Chit) was equilibrated within the first 20 ns of the refinement and it had 90–91% residues in a favorable conformation with just one nonglycine residue in a disallowed region of the Ramachandran plot (Figure S2A), the stability of the model was determined by the RMSD (root-mean-square deviation, Figure S2B) and RMSF (root-mean-square fluctuation, Figure S2C) plots. The active-site residues of *Tf*Chit were identified based on the homology relative to the template crystal structures (Figure S3). Catalytic residues are represented by D136 (transition state (TS) stabilizing residue) and E138 (acid/base residue), which are a part of the conserved catalytic motif of the GH18 chitinases D^134^ × D^136^ × E^138^. Despite the conserved catalytic residues, *Tf*Chit has differences in 2 loops close to the active site with highly homologous fungal chitinases from *A. fumigatus* and *A. niger* (Figure S3), where the loop W^209^–Q^211^ covering the +2 and +3 subsites is not conserved. The loop 348–358 of *Tf*Chit, close to the −3 subsite of the carbohydrate-binding cleft is longer than in homologous fungal chitinases and similar in length to chitinases from *Bacillus thuringiensis* (6bt9),^41^*O. furnacalis* (5wvb)^30^ and *Homo sapiens* (1guv).^42^ It was predicted^43^ and modeled as a coil corresponding to the loop found in *O. furnacalis* chitinase (5wvb). The modeled loop preserved its conformation during MD simulation (Figure S4).

**Figure 2:**
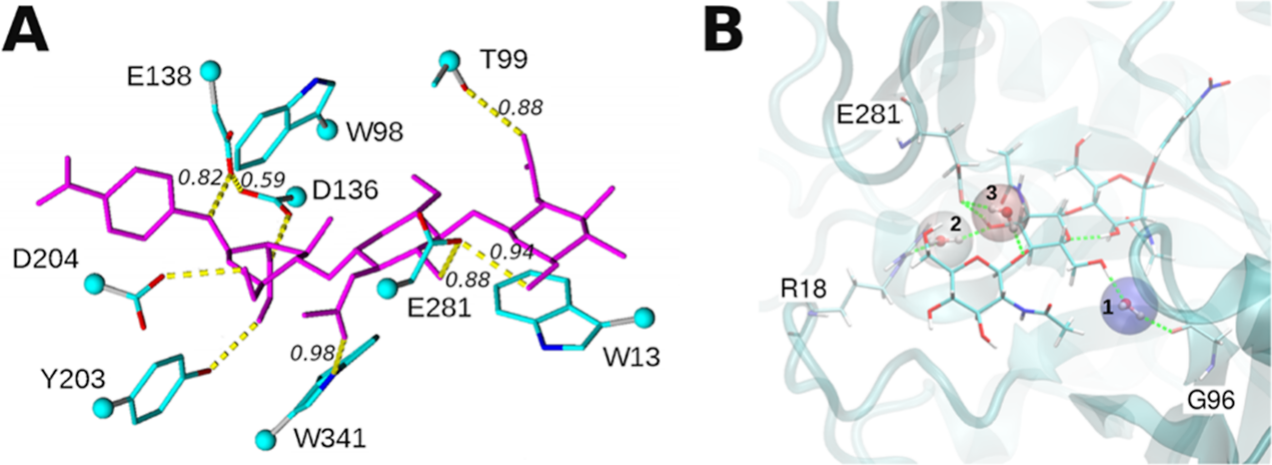
(A) *p*NP-CH3 docked in the active site of WT *Tf*Chit (after 100 ns of MD simulation). Hydrogen bonds formed during MD simulations with *p*NP-CH3 are shown as yellow dashed lines. Frequencies of the formation of hydrogen bonds during all 40–50 ns of MD are shown in *italics* if different from 1. The backbone is hidden, and the ligand is shown in magenta. (B) Water binding sites identified from MD simulations of the WT *Tf*Chit with *p*NP-CH3. The most populated site (1) is in blue, the least populated site in red (3): WFP-water finding probability is 28.8 for site (1); 13.63 for (2); and 11.9 for site (3).

The stability of enzyme–substrate complexes with *p*NP-CH3 and CH6 was analyzed during MD simulations with YASARA (Figures S5 and S6). The protein in the complex WT *Tf*Chit-*p*NP-CH3 was stabilized during the first 10 ns of MD (Figure S5). The stabilization of the ligand required more time, which is mainly due to the flexibility of the *p*-nitrophenyl group of the ligand (Figure S6). The most stable binding of the carbohydrate of CH6 occurs at the −1 subsite and the least stable is the carbohydrate bound at the −3 subsite. The number of hydrogen bonds (HB) formed by the *p*NP-CH3 ligand with the WT *Tf*Chit was stable during MD, the formed HBs are shown in Figure 2A. At least 3 water-mediated interactions were identified for the complex (Figure 2B), mainly at the −2 carbohydrate-binding subsite. It is important to notice that both D136 and E138 residues are protonated during MD and form HB, and D134 gets deprotonated (Figure 2A). E138 also forms hydrogen bonds with the *N*-acetyl group of the ligand, and we could conclude that the role of D136 includes the proper positioning of the −1 carbohydrate and the stabilization of catalytic E138. The exact role of D134 in hydrolysis is not clear as no direct interaction with the substrate or catalytic residues was detected during MD simulations of WT-*Tf*Chit.

**Figure 3:**
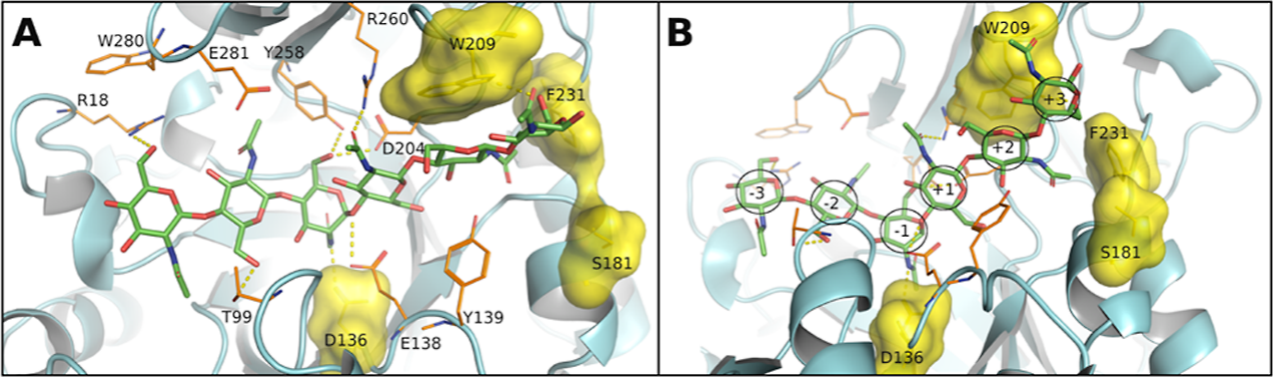
Hexasaccharide (CH6) substrate docked in the active site of *Tf*Chit. The mutation hotspots are shown as a yellow surface from the top (A) and side (B) view. Amino acid residues of WT *Tf*Chit, which could form HBs during MD with CH6, are shown and labeled; residues W98, W341, and Y203 as well as hydrogens are hidden for clarity.

The aromatic residues improved the substrate binding by stacking interactions. The *p*-nitrophenyl moiety of *p*NP-CH3 established a stacking interaction with W98 residue, however, it was unstable during MD simulations. The carbohydrate at the −3 subsite formed CH/π stacking interactions with W13 residue, which were preserved during MD. Other residues participating in the binding of longer chitooligomers were identified based on the MD simulations with the CH6 substrate. Four amino acid residues suitable for mutagenesis were identified (Figure 3): catalytic D136 and W209, S181, and F231 located at the +2 and +3 binding sites, respectively. Binding scores after MD simulation and average parameters of equilibrated chitinase-ligand (CH6, *p*NP-CH3) complexes are given in the Supporting Information, Table S3.

Molecular modeling and docking helped to understand the interactions in the active site of *Tf*Chit mutants with the substrates. Mutations in the active site of *Tf*Chit showed subtle changes in the binding of the −1 and +1 carbohydrate moieties, preserving functionality and activity. The D136V variant exhibited increased hydrolytic activity, attributed to improved interactions with the CH6 substrate, ligand shifts in the active site, and altered interactions at the +1 subsite (mainly flip of W98, Figure S7). The residue W209 placed between the +1 and +2 subsites (Figure 3B) is not strictly conserved in homologous chitinases and could be substituted by aromatic phenylalanine. The W209A mutation allowed the formation of new hydrogen bonds in the +3 subsite, facilitating easier binding of long carbohydrates for hydrolysis by providing a more hydrophobic environment.^44^

The extension of the active site by the S181W mutation should promote the binding of longer substrates. In contrast, it resulted in lower free energy of binding with CH6 and a complete loss of HB interaction with the carbohydrate in the +3 subsite, which had a negative effect on the binding of longer COS at the +3 subsite. Importantly, this mutation impaired the binding of the +3 carbohydrate moiety in all double mutants with the S181W modification, which may explain the lower hydrolytic activity of these variants with chitin (Table 2).

The F231W residue caused significant changes in interaction frequencies and hydrogen bond networks, affecting CH6 binding at the +3 subsite. The improvement of CH6 interaction at the +3 subsite compared to the WT enzyme may be responsible for an enhanced activity toward longer COS (Figure S8). Double/triple mutants exhibited altered hydrogen bonding networks that affect the increase in ligand flexibility and interactions (Figure S8). The S181W/D136V variant showed the lowest chitin hydrolysis activity due to lost hydrogen bonds of the substrate with the catalytic residues of the enzyme.

**Figure 4:**
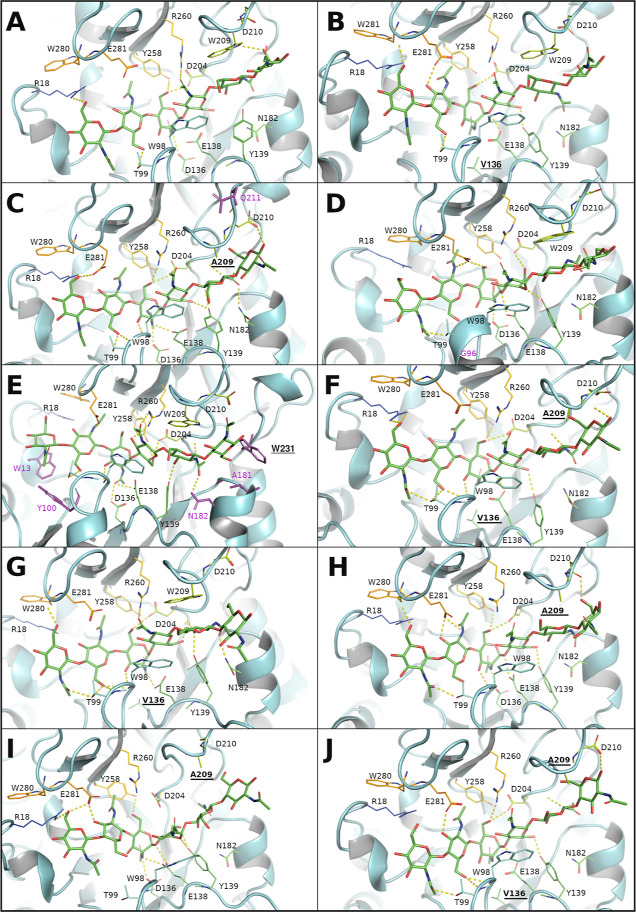
CH6 docked in the active site of *Tf*Chit variants after 50 ns of MD simulation; residues W341 and Y203 are hidden for clarity. Mutated residues are labeled in **bold** and underlined, magenta indicates the residues forming new hydrogen bonds with substrate only in the shown mutant. Hydrogen bonds are in yellow, the ligand is colored in element colors, and other residues are in the rainbow coloring scheme of the Pymol program. (A) WT *Tf*Chit; (B) D136V *Tf*Chit; (C) W209A *Tf*Chit; (D) S181W *Tf*Chit; (E) F231W *Tf*Chit; (F) D136V/W209A *Tf*Chit; (G) S181W/D136V *Tf*Chit; (H) W209A/F231W *Tf*Chit; (I) S181W/W209A *Tf*Chit; (J) S181W/W209A/D136V *Tf*Chit.

Figure 4 provides a detailed insight into the binding of the CH6 substrate in the active site of *Tf*Chit and its mutant variants. While no significant reorientation of the active site residues was identified, numerous small changes, particularly in the double/triple mutants, rendered them less effective than the individual mutants. Dual mutation at the +3 subsite caused rotation of the +3 carbohydrate in S181W/W209A and S181W/W209A/D136V (Figures 4I,J and S9), but the active site mutation D136V allowed deeper placement of CH6 in the −1/–2 sites of the triple mutant and formation of HB interactions by the +3 carbohydrate (Figures S9 and S10). The CH6 substrate showed stable binding in these mutants but with a slightly higher free energy of binding (i.e., weaker binding) than in the single mutants (Figure 4). Combined mutations had a generally negative effect, leading to the distortion of interactions of the substrate with active-site residues.

### Synthesis of Insoluble Chitooligomers Using Mutant β-*N*-Acetylhexosaminidase from *A. oryzae* (*Ao*Hex)

**Figure 5:**
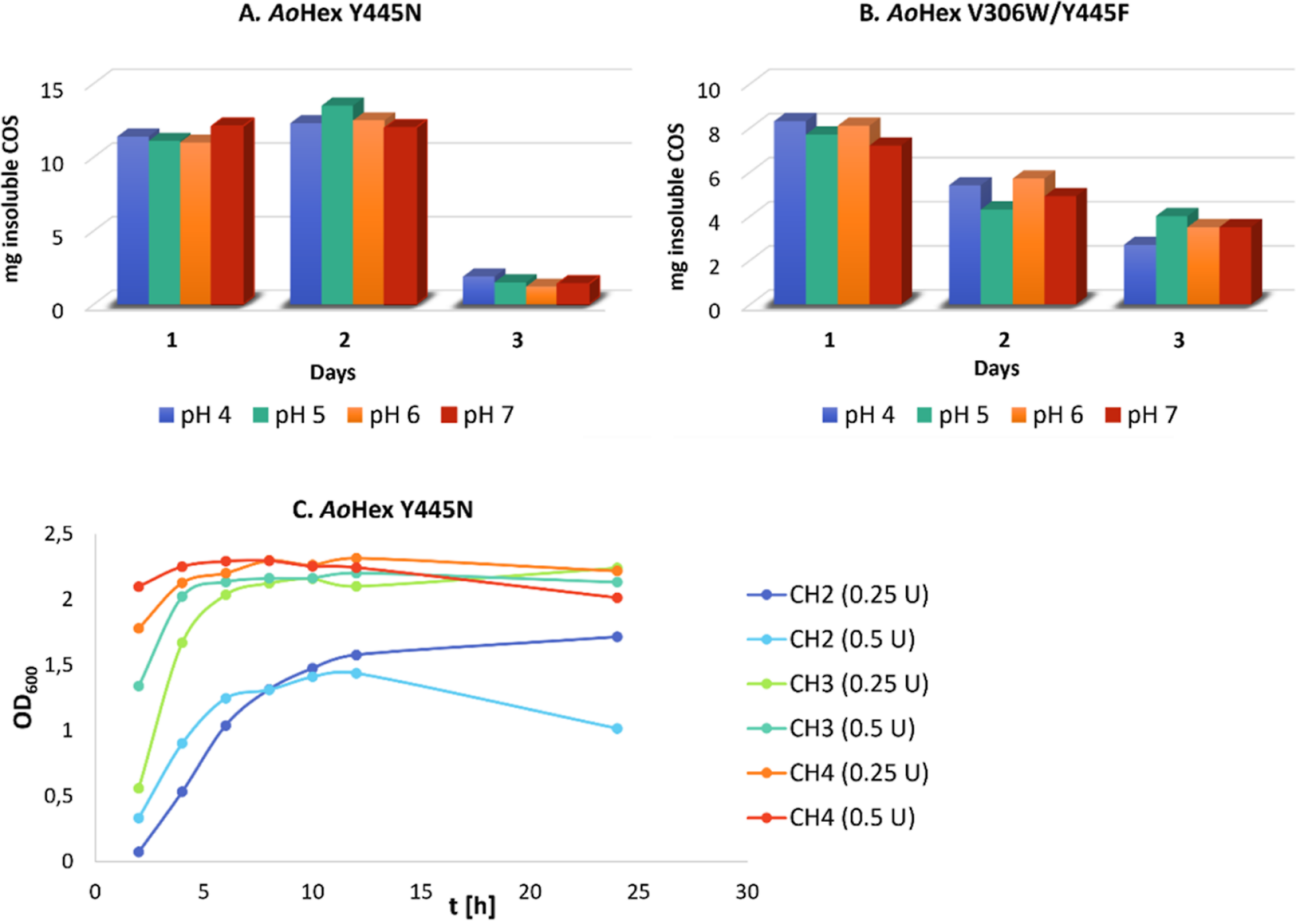
Plots of weight yields [mg] of insoluble COS prepared using mutant variants of β-*N*-acetylhexosaminidase from *Aspergillus oryzae* (*Ao*Hex) with CH3 (100 mM) as a substrate. (A) Y445N *Ao*Hex; (B) V306W/Y445F *Ao*Hex; (C) synthesis of insoluble chitooligomers catalyzed by Y445N *Ao*Hex in two concentrations using substrates CH2, CH3, and CH4. OD_600_ describes the turbidity of the reaction suspension, approximately proportional to the amount of the formed insoluble COS.

In the previous transglycosylation reactions with engineered *Ao*Hex variants, the formation of insoluble chitooligomers with DP ≥ 6 was observed in Y445N and V306W/Y445F variants.^13^ This study focused on the determination of their ability to synthesize the target insoluble chitooligomers using defined low-DP COS (CH2, CH3, and CH4) as substrates. In the initial experiments, Y445N or V306W/Y445F *Ao*Hex variants and 30 mg of CH3 substrate were used in the pH range from 4 to 7. Reactions were supplemented with 15 mg of CH3 every 24 h, and solid products were isolated by centrifugation. Yields, shown in Figure 5, revealed that the Y445N variant (Figure 5A) was significantly more effective in synthesizing higher-DP chitooligomers than the double mutant V306W/Y445F (Figure 5B), which we attribute to the higher residual hydrolytic activity of the latter. The Y445N variant afforded a maximum weight of 13.5 mg of insoluble COS after 48 h at pH 5.

The impact of pH on the reaction yields was minimal with both enzyme variants; therefore, pH 5 was selected for further experiments with the more efficient enzyme variant. Subsequent investigations focused on determining the optimum enzyme amount and substrate length to achieve maximum yields of high-DP COS. Transglycosylation reactions utilized defined chitooligomers with DP 2, 3, or 4 as substrates, employing Y445N *Ao*Hex at two concentrations on an analytical scale. The progress of the reaction was monitored by spectrophotometric determination of turbidity (optical density, OD) at 600 nm. The results revealed that CH3 and CH4 were superior substrates compared to CH2, reaching maximum measurable OD values after 6 h (Figure 5C). Semipreparative scale reactions were then conducted, affording mixtures of insoluble COS. The highest yields were obtained using 0.5 U of enzyme per reaction, yielding approximately 13 mg of insoluble COS (43% *w*/*w*) from 30 mg of CH3 after 24 h and about 17 mg (57% *w*/*w*) when CH4 was the substrate (Table 3). The composition of the mixture of solid chitooligomers was determined by MALDI-TOF mass spectrometry, indicating that COS with DP from 6 to 11 were produced in these transglycosylation reactions (Figure S11).

**Table 3:** Isolated Weight Yields of Insoluble COS Afforded from 30 mg CH2/CH3/CH4 (100 mM) Using the Y445N Variant of β-*N*-Acetylhexosaminidase from *Aspergillus oryzae* (*Ao*Hex) After a 24 h Reaction

substrate	enzyme [U]	insoluble COS [mg]	yields [%]
CH2	0.5	5.6	19
CH3	0.25	11.1	37
CH3	0.5	12.9	43
CH3	1	10.9	36
CH4	0.5	17.1	57

Comparisons with a related engineered variant of *Tf*Hex (Y470N) showed that Y445N *Ao*Hex could produce insoluble COS with higher DP. It reaches DP 11 compared to DP 7 observed in the reactions catalyzed by *Tf*Hex.^14^ Thus, the easily scalable production of higher-DP COS comprising up to 11 GlcNAc units is unique in β-*N*-acetylhexosaminidase-catalyzed transglycosylation reactions. Alternatively, higher-DP COS can be synthesized in transglycosylation reactions employing engineered chitinases, however, these enzymes require the less available (and more expensive) substrates with DP of 4–5 or even their oxazoline forms.^45,46^ Our results highlight an outstanding enzymatic method of an efficient synthesis of chitooligomers of 6 to 11 GlcNAc units using chitobiose and chitotriose as substrates, which are typical predominant products of chitinase-assisted hydrolysis of chitin. Transglycosylation reactions employing the Y445N *Ao*Hex have a strong potential for seamless integration into a highly effective, large-scale biotechnology process, utilizing and upgrading low-DP COS from the abundant chitin waste from the food industry.

### Advanced Enzymatic Deacetylation of Unique Chitooligomers

**Figure 6:**
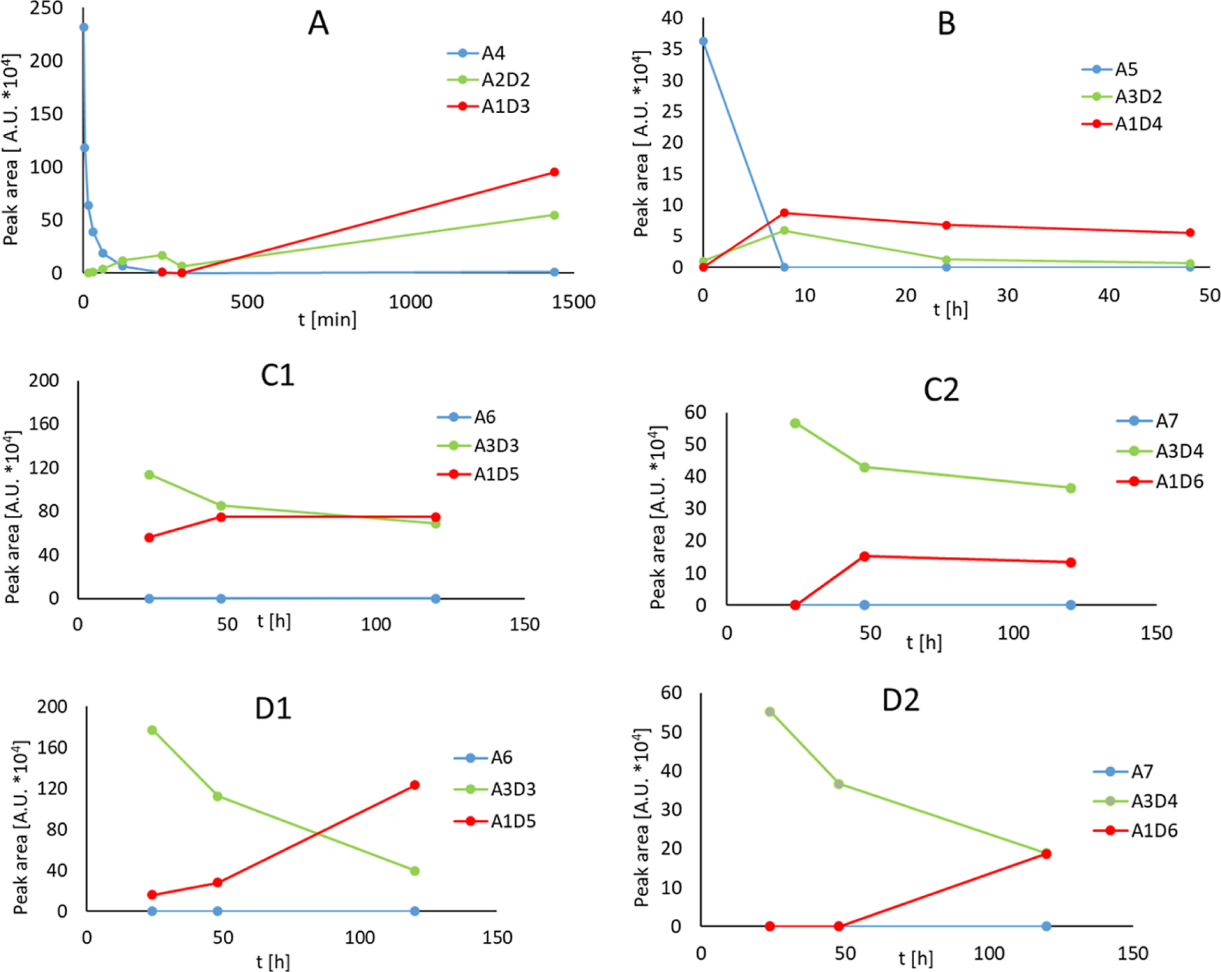
Progress of deacetylation reactions using enzyme *Bs*PdaC and monitored by HPLC-MS. (A) Substrate CH4 (2 mM) and 4 μM enzyme (A4: fully acetylated CH4, A2D2: partially deacetylated CH4, A1D3: fully deacetylated CH4). (B) Substrate CH5 (2 mM) and 4 μM enzyme (A5: fully acetylated CH5, A3D2: partially deacetylated CH5, A1D4: fully deacetylated CH5). (C1) Insoluble COS (10 mg) analysis of CH6, 20 μM enzyme, 10 mM NH_4_HCO_3_ (A6: fully acetylated CH6, A3D3: partially deacetylated CH6, A1D5: fully deacetylated CH6). (C2) Insoluble COS (10 mg) analysis of CH7, 20 μM enzyme, 10 mM NH_4_HCO_3_ (A7: fully acetylated CH7, A3D4: partially deacetylated CH7, A1D6: fully deacetylated CH7). (D1) Insoluble COS (10 mg) analysis of CH6, 20 μM enzyme, 0.5% Triton X-100. (D2) Insoluble COS (10 mg) analysis of CH7, 20 μM enzyme, 0.5% Triton X-100.

In this study, we conducted pilot experiments on the enzymatic deacetylation of insoluble chitooligomers produced by *Ao*Hex Y445N. Notably, deacetylation of such higher insoluble COS is unprecedented in the literature, as our COS samples are truly unique.^47^ We employed peptidoglycan deacetylase from *B. subtilis* (*Bs*PdaC) for this enzymatic deacetylation. Chitin and peptidoglycan deacetylases belong to the same enzyme family (CAZY CE4 family).^48^*Bs*PdaC has previously been shown to efficiently deacetylate COS from DP3 to 5, deacetylating all units except the reducing end GlcNAc unit and following a mechanism of multiple binding (distributive) by partially deacetylated intermediates (i.e., A5 → A4D1 → A3D2 → A2D3 → A1D4, being A = GlcNAc and D = GlcN units).^36,48^ Since peptidoglycan deacetylases act on insoluble peptidoglycan, we speculated that they might also act on insoluble COS. We selected *Bs*PdaC due to its high activity on both insoluble peptidoglycan and soluble COS.^20^ Deacetylation reactions were performed by adjusting concentrations and introducing additives (NH_4_HCO_3_, Triton X-100) to enhance reactions with insoluble COS. Figure 6 illustrates the deacetylation progress catalyzed by *Bs*PdaC, monitored using HPLC-MS. The fully deacetylated (final A1D(n-1) products) and selected partially deacetylated chitooligomers were analyzed based on the *m*/*z* values monitored by HPLC-MS (Table S4).

*Bs*PdaC exhibited an effective deacetylation of standard CH4 and CH5 substrates, producing partially and fully deacetylated COS in approximately 10 h (Figure 6A,B). Encouraged by these results, subsequent tests employed insoluble COS mixtures with DP 6–11 as substrates obtained from the TG reactions by *Ao*Hex Y445N. Interestingly, COS with DP 6 and 7 exhibited successful partial and full deacetylation by *Bs*PdaC (Figure 6C,D). However, products of DP ≥ 8 resisted deacetylation, despite enrichment of the reactions with 10 mM NH_4_HCO_3_ or 0.5% Triton X-100 to enhance enzyme-insoluble COS interaction. These pivotal experiments on enzymatic deacetylation uncover a significant potential of this deacetylase and highlight future challenges in the enzymatic processing of insoluble chitooligomers.

In this study, a three-step enzymatic pathway for the efficient and sustainable conversion of chitin-containing waste from the food industry into desirable chitooligomers with a DP of 6 to 11 is presented. First, the engineered variants of a novel chitinase hydrolyzed chitin and produced chitooligomers with low DP. The Y445N variant of a fungal β-*N*-acetylhexosaminidase extended the low-DP chitooligomers to higher DP (6–11), achieving yields of up to 57% of the target COS products. Moreover, successful enzymatic deacetylation of COS with DP 6 and 7 was achieved using the peptidoglycan deacetylase *Bs*PdaC.

The current setting of the three-step enzymatic pathway is the proof of concept of each step. In practice and future work to implement a fully sequential process, the products from chitin degradation by chitinase would have to be partially purified to remove salts and concentrate them up to the required concentration for the next transglycosylation reaction by β-*N*-acetylhexosaminidase. The deacetylation step has already been applied to the insoluble COS (DP 6–11) fraction from the transglycosylation reaction. This innovative approach enables the sustainable production of valuable chitooligomers from chitin-rich waste.

## Supporting Information Available

PCR primers and reaction conditions for the generation of *Tf*Chit mutants; HPLC chromatogram of chitin hydrolysate; detailed sequence alignment; computational data; additional figures for modeling of *Tf*Chit; MALDI-TOF MS analysis of a mixture of insoluble chitooligomers prepared by Y445N *Ao*Hex, and *m*/*z* values monitored by HPLC-MS of the deacetylation reactions (PDF)
