# Supplementary material for: Three-Step Enzymatic Remodeling of Chitin into Bioactive Chitooligomers
Source: J Agric Food Chem. 2024 Jul 9;72(28):15613–23. doi: 10.1021/acs.jafc.4c03077 (PMC11261597; doi:10.1021/acs.jafc.4c03077)
Supplement: Supplementary file 1 — jf4c03077_si_001.pdf [file jf4c03077_si_001.pdf]

Supporting Information

Three-step enzymatic remodeling of chitin into bioactive chitooligomers

Zuzana Mészáros[a], Natalia Kulik[a], Lucie Petrásková[a], Pavla Bojarová [a], Mònica Texidó[b], Antoni Planas[b], Vladimír Křen[a], Kristýna Slámová[a, *]

[a] *Institute of Microbiology of the Czech Academy of Sciences, Vídeňská 1083, Prague 4, CZ 14200, Czech Republic*

[b]*Laboratory of Biochemistry, Institut Químic de Sarrià, University Ramon Llull, ES 08017 Barcelona, Spain*

** Corresponding author*

e-mail: slamova@biomed.cas.cz

Table S1. Primers for the site-directed mutagenesis of *Tf*Chit and annealing temperature T_a used in the respective PCR.

*Tf*Chit Mutant	Primers	T_a [°C]
**D136V**[a]	Fw 5´-TTGATGGAATTGATATTGTTTGGGAATACC-3´<br>Re 5´-GGTATTCCCAAACAATATCAATTCCATCAA -3´	47
**W209A**[a]	Fw 5´- TACGCCGGTTCAGCGGATCAAGTTGCTGGA-3´<br>Re 5´- TCCAGCAACTTGATCCGCTGAACCGGCGTA -3´	61
**S181W**[a]	Fw 5´- GCTGGAGCCTGGAACTACCAAAAGCTACGT -3´<br>Re 5´- ACGTAGCTTTTGGTAGTTCCAGGCTCCAGC -3´	57
**F231W**[a]	Fw 5´-CGCTTCTGCCACCCCATGGTCTACCGTTGG -3´<br>Re 5´-CCAACGGTAGACCATGGGGTGGCAGAAGCG -3´	63
**W209A/D136V**[b]	Fw 5´-TTGATGGAATTGATATTGTTTGGGAATACC-3´<br>Re 5´-GGTATTCCCAAACAATATCAATTCCATCAA -3´	47
**W209A /F231W**[b]	Fw 5´-TTGATGGAATTGATATTGTTTGGGAATACC-3´<br>Re 5´-GGTATTCCCAAACAATATCAATTCCATCAA -3´	47
**S181W/D136V**[c]	Fw 5´-TTGATGGAATTGATATTGTTTGGGAATACC-3´<br>Re 5´-GGTATTCCCAAACAATATCAATTCCATCAA -3´	47
**S181W/ W209A** [c]	Fw 5´- TACGCCGGTTCAGCGGATCAAGTTGCTGGA-3´<br>Re 5´- TCCAGCAACTTGATCCGCTGAACCGGCGTA -3´	61
**S181W/ W209A / D136V**[d]	Fw 5´-TTGATGGAATTGATATTGTTTGGGAATACC-3´<br>Re 5´-GGTATTCCCAAACAATATCAATTCCATCAA -3´	47

Template plasmid: [a]WT *Tf*Chit, [b]W209A *Tf*Chit , [c]S181W *Tf*Chit, [d]S181W/W209A *Tf*Chit.

Table S2. Conditions for the PCR reaction catalyzed by *Pfu* Ultra high-fidelity DNA polymerase (QuikChange Lightning Site Directed Mutagenesis Kit).

Number of cycles	Temperature [°C]	Time [s]
1	95	120
18	95	20
	T_a	10
	68	180
1	68	300

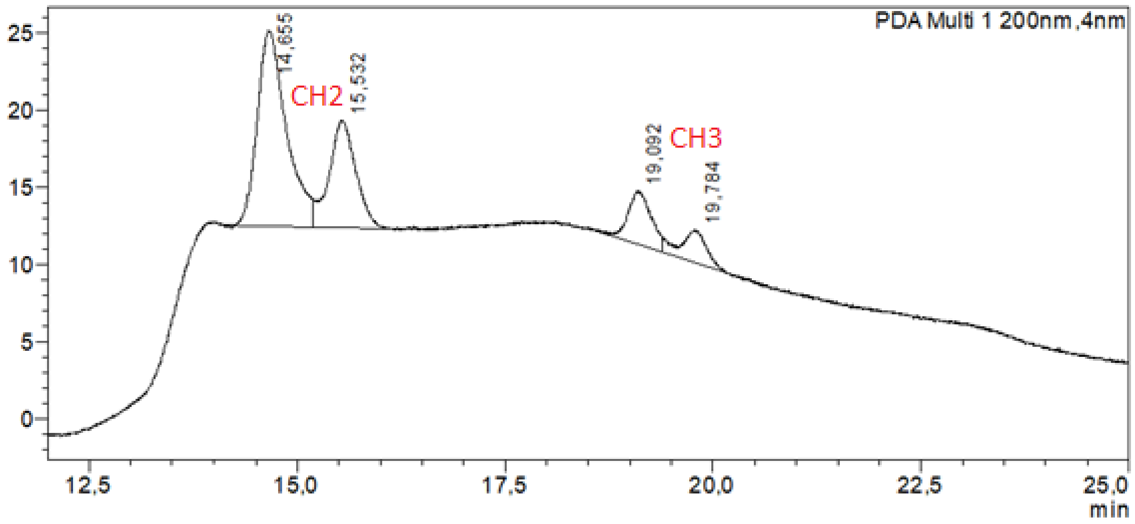

Figure S1. HPLC chromatogram of chitin hydrolysate prepared by mutant F231W chitinase from *Talaromyces flavus* (*Tf*Chit). The sample was measured on a HILIC column (TSKgel Amide-80), where anomer separation takes place. HPLC analyses were performed on a Shimadzu Prominence LC analytical system consisting of Shimadzu SIL-20ACHT cooling autosampler, Shimadzu LC-20AD binary HPLC pump, Shimadzu CTO-10AS column oven, Shimadzu SPD-20MA diode array detector and Shimadzu CBM20 A system controller and analyzed with software LabSolutions ver. 5.75 SP2 (Shimadzu, JP). The sample (20 µL) was dissolved in the mobile phase (acetonitrile/water = 80/20; 60 µL) and analyzed on TSKgel Amide-80, 5 µm HILIC column (250 × 4.6 mm, Tosoh Bioscience, JP) equipped with TSKgel guard column (15 × 3.2 mm, Tosoh Bioscience, JP). Binary gradient elution was used: mobile phase A = 100% acetonitrile; mobile phase B = water; gradient: 22% B for 0–7 min, 22–35% B for 7–20 min; 35% B for 20– 25 min, 35–22% B for 25–26 min. The flow rate was 1 mL/min at 25 °C, and the injection volume was 1 µL; samples were detected at 200 nm. Retention times of the analytes (min): CH2 (14.655, 15.532; α- and β-anomers, respectively); CH3 (19.092, 19.784; α- and β-anomers, respectively).

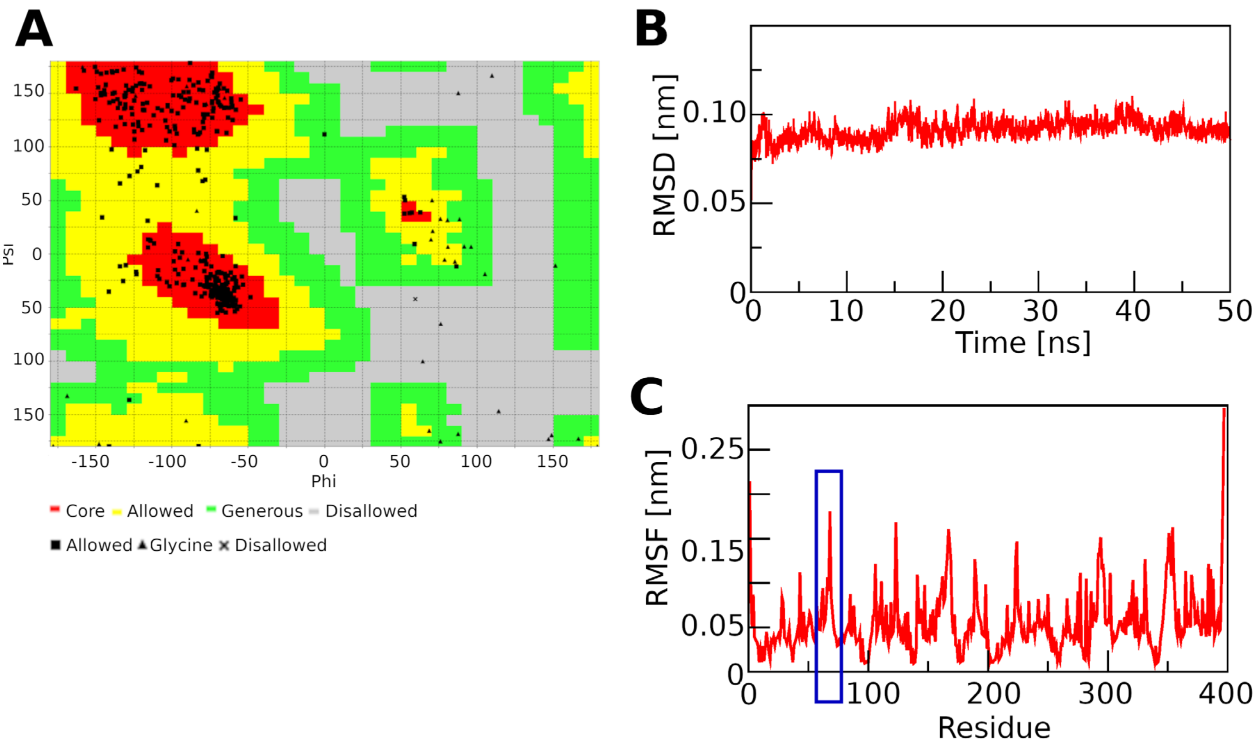

Figure S2. Statistics of homology modeling refinement (WT *Tf*Chit). A. Ramachandran plot made with Vadar after 50 ns of MD simulation. **B.** RMSD (root means square deviation) of protein C-alpha atoms during refinement. **C.** RMSF (root means square fluctuation) of the residues during 50 ns of MD simulation. The most flexible regions include loop 68-73, placed far from the active site, C- and N-terminals.

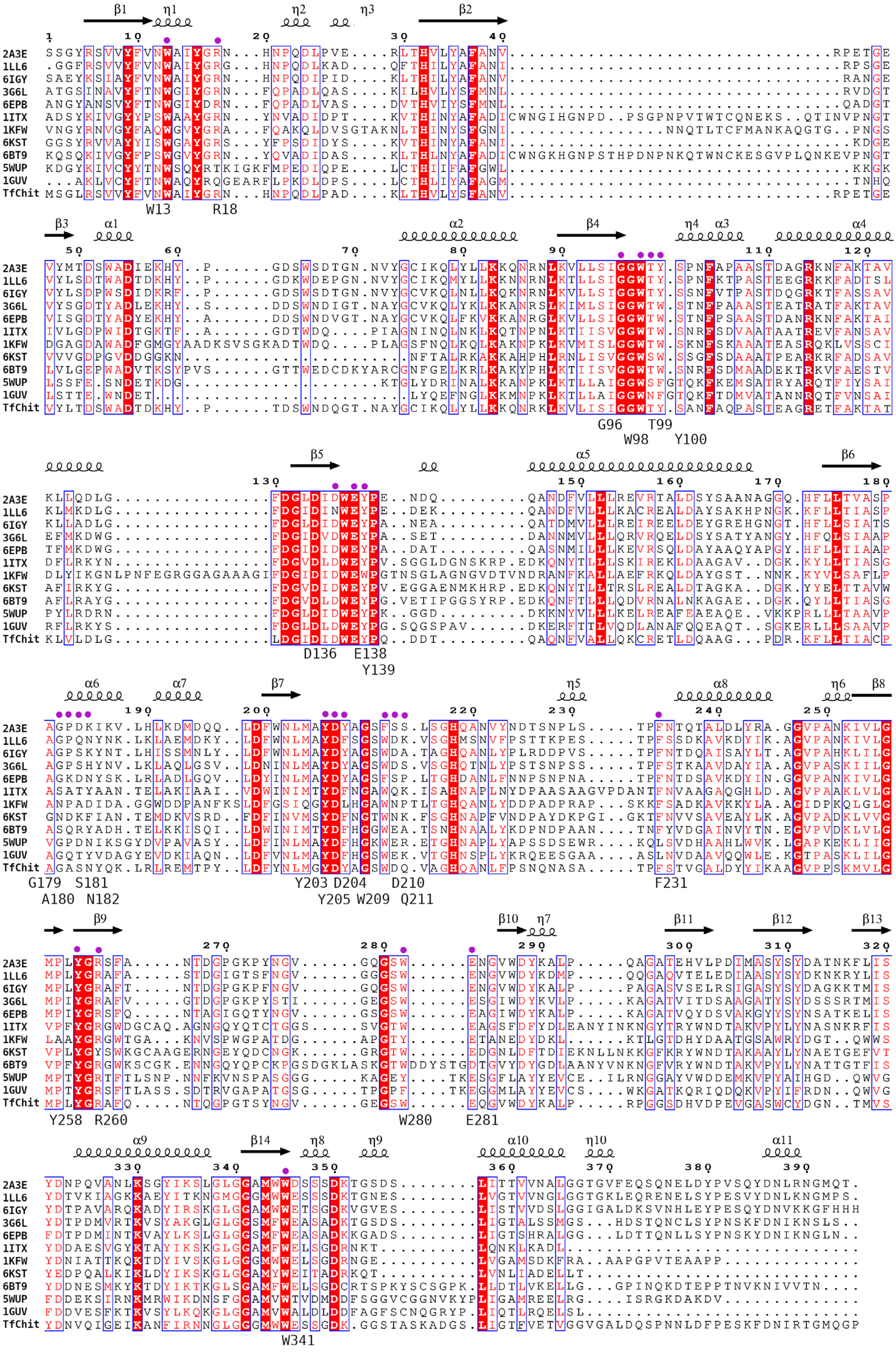

2A3E
1LL6
6IGY
3G6L
6EPB
1ITX
1KFW
6KST
6BT9
5WUP
1GUV
TfChit
W13
R18
G96
T99
W98
Y100
D136
E138
Y139
G179
S181
A180
N182
Y203
D204
D210
Y205
W209
Q211
F231
Y258
R260
W280
E281
W341

Figure S3. Multiple sequence alignment of homologs of *Tf*Chit. Representation was visualized by ESPript The secondary structure is assigned from the 2a3e structure, the residues discussed in the article are marked by magenta dots (numeration according to *Tf*Chit). The best homologs are from *Aspergillus fumigatus* (pdb: 2a3e, 61.9% identity, 99% coverage), *Coccidioides immitis* (pdb: 1ll6, 61.21% identity, 98% coverage), *Aspergillus niger* (pdb: 6igy, 58.73% identity, 98% coverage). Lower identities were found with chitinases from *Clonostachys rosea* (3g6l; 53.88%; 99%), *Trichoderma harzianum* (6epb; 51.88%, 99%), *Niallia circulans* (1itx; 32.45%, 89%), *Arthrobacter* sp. TAD20 (1kfw, 33.25%, 87%), *Chitiniphilus shinanonensis* (6kst; 32.97%; 87%); *Bacillus thuringiensis* (6bt9, 31.45%; 93%), human (1guv, 29.9%; 84%), and *Ostrinia furnacalis* (5wup, 30.15%, 92%). The loop 348-358 is close to the -3 carbohydrate subsite and has a different size in *Tf*Chit than in the closest fungal homolog from *Aspergillus fumigatus*.

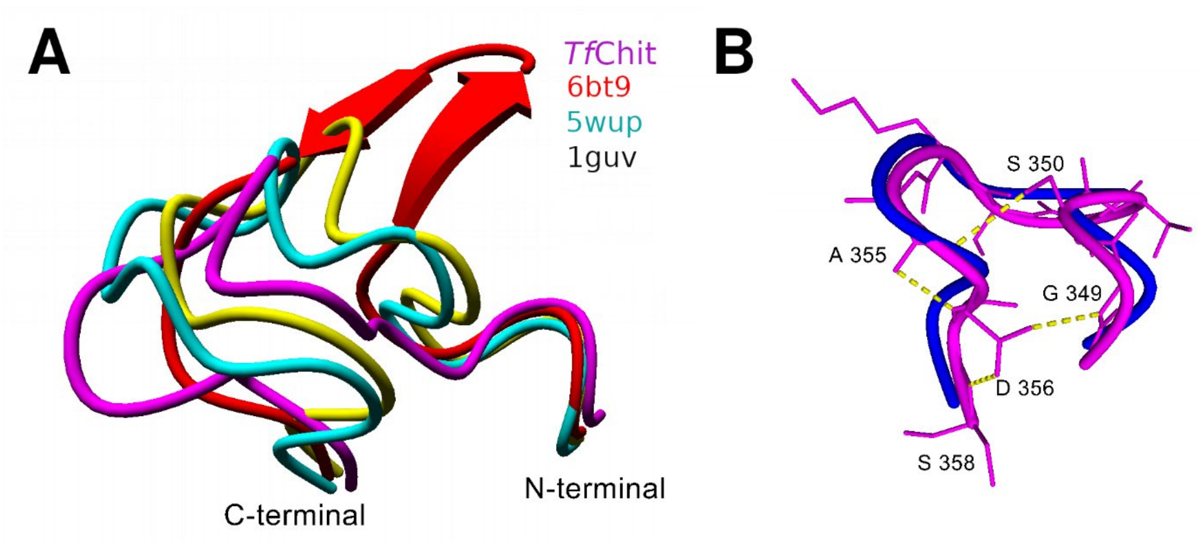

Figure S4. A. Comparison of loops corresponding to residues 348-358 in *Tf*Chit between pdb structures: a refined model of *Tf*Chit (magenta), bacterial chitinase from *Bacillus thuringiensis* (6bt9), the insect chitinase from *Ostrinia furnacalis* (5wup) and chitinase from *Homo sapiens* (1guv). **B.** Position of loop 348-358 before MD (blue) and after 50 ns of MD (magenta). HBs formed after 50 ns of MD simulation are shown: G349-D356, S350-D356, A355-G357, D356-S358.

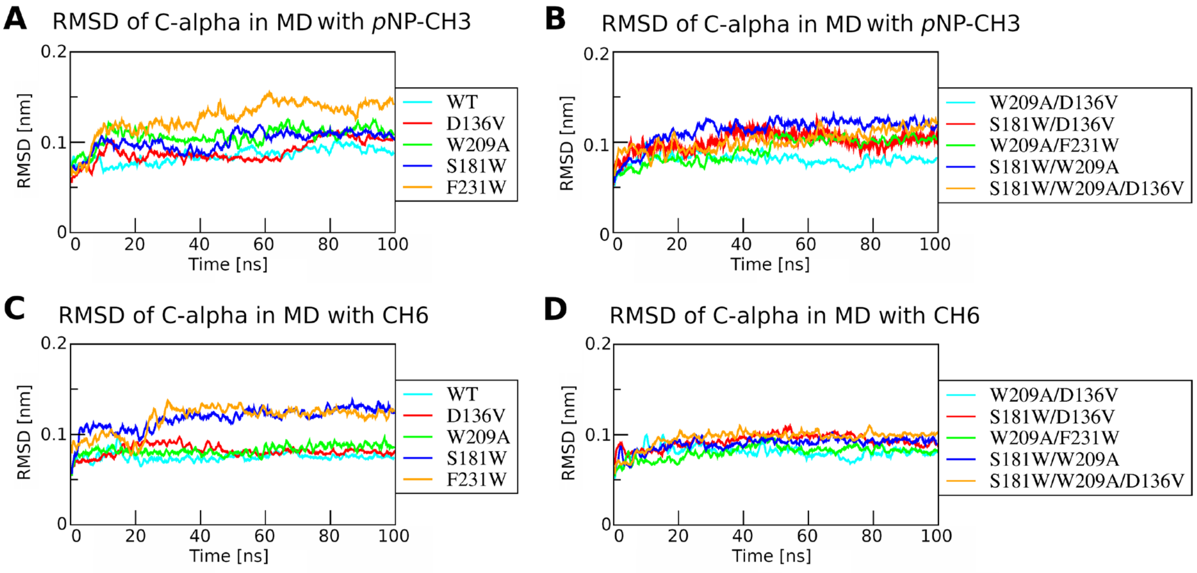

Figure S5. Root means square deviation (RMSD) of the protein C-alpha atoms of WT and mutant *Tf*Chit with docked different ligands. **A.** RMSD of C-alpha atoms with *p*NP-CH3 and WT and single mutants of *Tf*Chit. **B.** RMSD of C-alpha atoms with *p*NP-CH3 and double/triple mutants of *Tf*Chit. **C.** RMSD of C-alpha atoms with CH6 and WT and single mutants of *Tf*Chit. **D.** RMSD of C-alpha atoms with CH6 and double/triple mutants of *Tf*Chit.

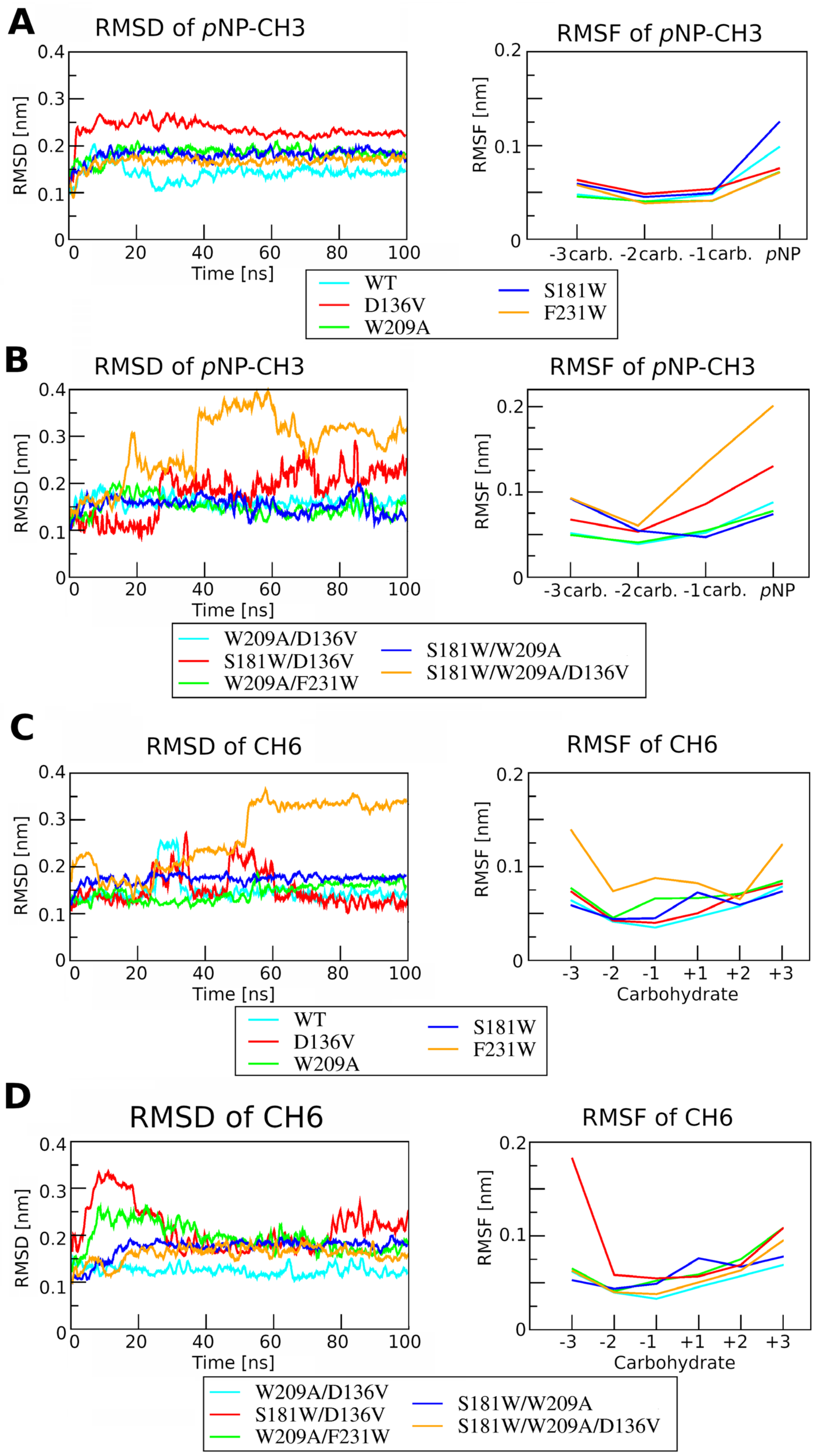

Figure S6. Root means square deviation (RMSD, left panel) and root means square fluctuation (RMSF, right panel) of ligands during MD simulation. **A.** RMSD and RMSF of *p*NP-CH3 and WT and single mutants of *Tf*Chit. **B.** RMSD and RMSF of *p*NP-CH3 and double/triple mutants

of *Tf*Chit. **C.** RMSD and RMSF of CH6 and WT and single mutants of *Tf*Chit. **D.** RMSD and RMSF of CH6 and double/triple mutants of *Tf*Chit.

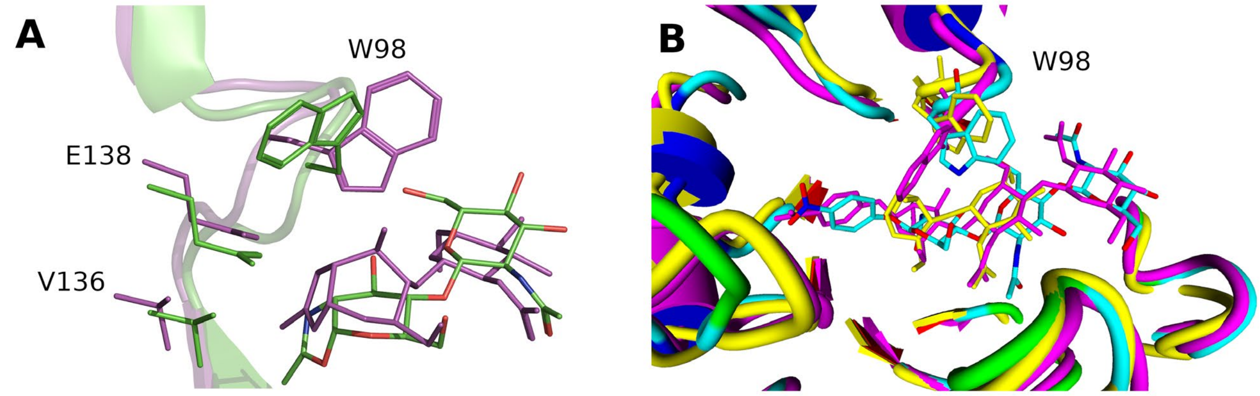

Figure S7. A. D136V mutant variant with the structure of CH2-oxazoline reaction intermediate, overlay of 0 ns (enzyme in green, intermediate in element colors) and 100 ns (magenta color). **B.** Overlay of equilibrated complexes of W209A-*p*NP-CH3 (element colors), WT-*p*NP-CH3 (magenta), and D136V-oxazoline intermediate with shown change in the W98 orientation (yellow).

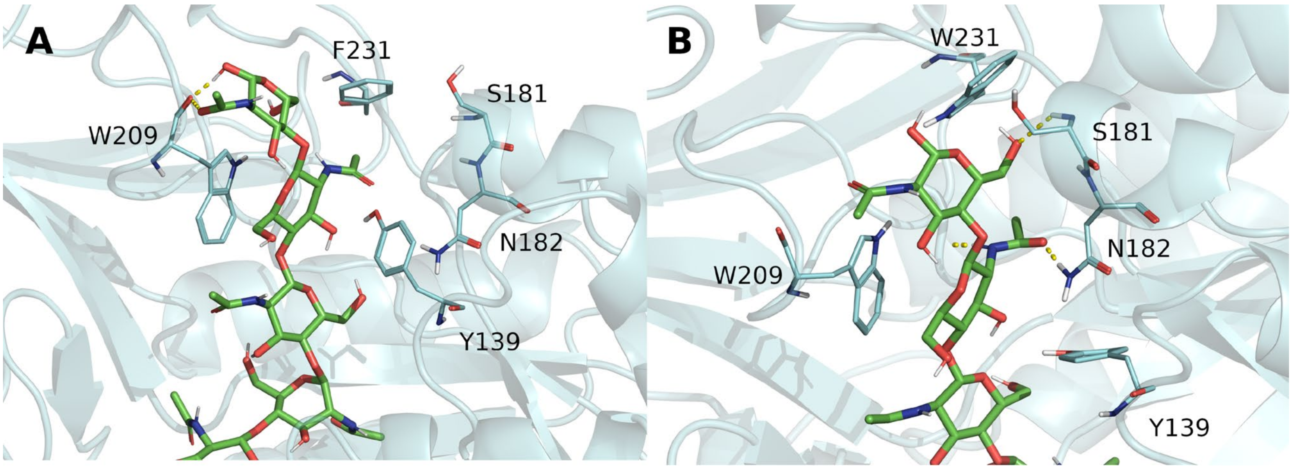

Figure S8. Interactions of the +2 and +3 carbohydrates of CH6 after 100 ns of MD simulation in the active site of **A.** WT *Tf*Chit and **B.** F231W mutant variant. Non-polar hydrogens are hidden. HBs are shown by yellow dashed lines.

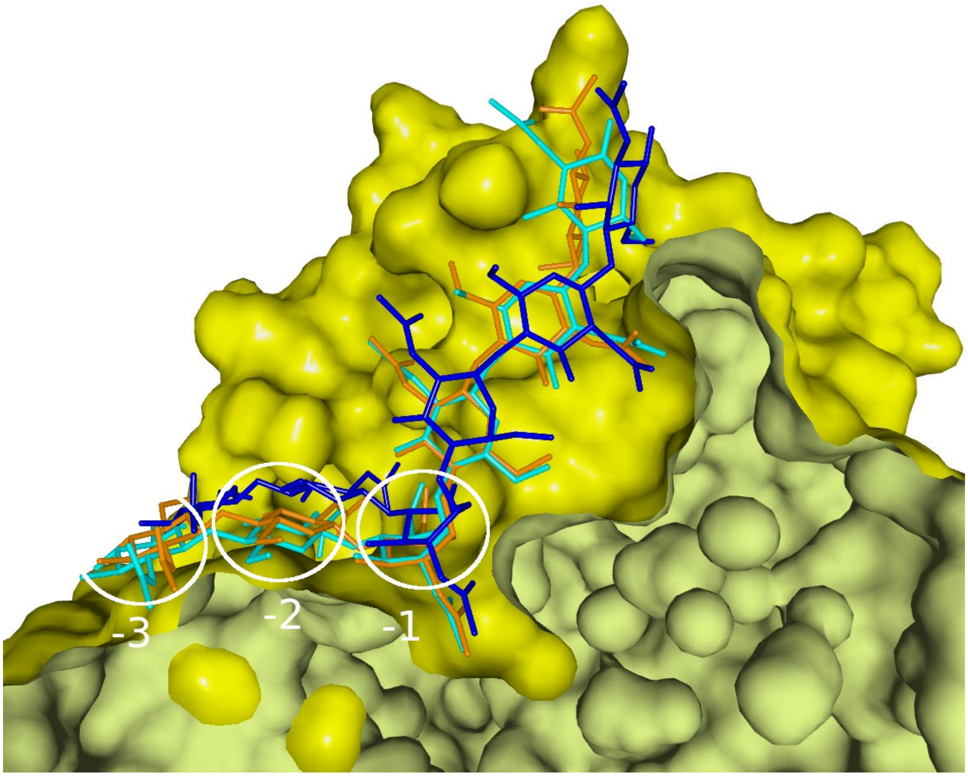

Figure S9. Orientation of the CH6 interpolated by alignment of the WT (cyan), S181W/W209A (blue), and S181W/W209A/D136V (orange) with docked CH6 after 50 ns MD simulation. Protein is shown by the cut protein surface (structure S181W/W209A was used for visualization, inner protein pace is in pale yellow). Subsites are labeled.

A.

HB	enzyme residue	WT	D136V	W209A	S181W	F231W	S181W/ W209A/ D136V	W209A/ D136V	S181W/ D136V	W209A/ F231W	S181W/ W209A
' - 3'	GLU281	0.94	0.72	0.00	0.00	0.00	0.00	0.04	0.44	0.90	0.33
	THR99	0.88	0.16	0.00	0.28	0.00	0.56	0.21	0.86	0.00	0.15
	TRP280	0.04	0.10	0.00	0.00	0.00	0.06	0.39	0.00	0.02	0.14
	ARG18	0.00	0.86	0.24	0.62	0.00	0.25	0.08	0.60	0.90	0.42
	TRP98	0.00	0.00	0.28	0.00	0.00	0.00	0.00	0.00	0.00	0.00
' - 2'	TRP341	0.98	1.00	1.00	0.94	0.90	0.00	1.00	0.94	0.98	0.50
	GLU281	0.88	0.92	1.00	0.98	0.96	0.14	1.00	0.35	0.79	0.00
	THR99	0.72	0.14	0.26	0.30	1.00	0.17	1.00	0.66	0.98	0.14
	TRP98	0.02	0.26	0.02	0.08	0.08	0.10	0.08	0.42	0.21	0.25
	ARG18	0.00	0.00	0.00	0.14	0.00	0.00	0.00	0.00	0.02	0.00
	TRP280	0.00	0.00	0.06	0.26	0.06	0.98	0.00	0.00	0.00	0.50
' - 1'	TYR203	1.00	1.00	0.98	1.00	1.00	0.00	1.00	0.96	1.00	0.54
	ASP204	1.00	0.96	0.00	0.00	0.00	0.00	1.00	0.00	0.04	0.00
	**res136**	1.00	0.00	0.98	0.46	1.00	0.00	0.00	0.00	1.00	0.54
	TRP98	0.82	0.62	0.74	0.00	0.78	0.00	0.94	0.28	0.69	0.23
	ARG260	0.06	0.26	0.00	0.06	0.00	1.00	0.64	0.00	0.42	0.04
	GLU138	0.00	0.00	0.00	0.04	0.00	1.00	0.31	0.23	0.00	0.00
	TYR258	0.00	0.00	0.00	0.00	0.00	0.08	0.00	0.00	0.00	0.02
	GLU281	0.00	0.00	0.98	0.98	0.44	1.00	0.00	0.00	0.00	0.10
***p*NP**	GLU138	0.82	0.00	0.86	0.66	0.98	0.00	1.00	0.94	0.98	0.54
	TYR139	0.00	0.00	0.00	0.00	0.00	0.00	0.15	0.00	0.06	0.00
	ASN182	0.00	0.00	0.00	0.00	0.00	0.00	0.00	0.10	0.00	0.00
**Total**		9.14	7.40	7.92	7.70	7.20	6.19	9.46	7.80	9.02	4.81

Pi-Pi/ Cation-Pi	enzyme residue	WT	D136V	W209A	S181W	F231W	S181W/ W209A/ D136V	W209A/ D136V	S181W/ D136V	W209A/ F231W	S181W/ W209A
*p*NP	TRP98	0.00	0.00	0.00	0.00	0.00	0.84	0.00	0.00	0.00	0.00
	TYR139	0.90	0.00	0.92	0.94	0.82	0.00	0.72	0.72	0.88	0.36
	ARG260	0.00	0.00	0.02	0.22	0.00	0.00	0.00	0.00	0.00	0.00

B.

HB	enzyme residue	WT	D136V	W209A	S181W	F231W	S181W/ W209A/ D136V	W209A/ D136V	S181W/ D136V	W209A/ F231W	S181W/ W209A
' - 3'	ARG18	0.94	0.08	0.24	0	0.02	0.26	0.72	0	0.02	0.94
	GLU281	0.28	0.04	0.64	0	0	0.04	0.12	0	0	0.92
	THR99	0.08	0.26	0.2	0.8	0.22	0.88	0.32	0.76	0.98	0
	TRP280	0.02	0.34	0.02	0	0	0.14	0.04	0.94	0.2	0
	TYR100	0	0	0	0	0.18	0	0	0	0	0
' - 2'	THR99	1	0	1	0.86	0.86	0.78	1	1	0.88	0
	TRP341	0.94	0.98	0.86	0.7	0	0.98	1	0.98	1	0.94
	GLU281	0.38	0.94	0.52	0.96	0.26	0.66	0.16	0.38	0.96	1
	TRP98	0.02	1	0	0	0.68	0	0	0.02	0	0
	TRP13	0	0	0	0	0.04	0	0	0	0	0
	ARG18	0	0	0	0	0.02	0	0	0.02	0	0
	GLY96	0	0	0	0.02	0	0	0	0	0	0
	TRP280	0	0	0	0.02	0	0	0	0	0	0
	TYR100	0	0	0	0	0.12	0	0	0	0	0
' - 1'	TRP98	1	0.9	1	0.9	0.96	0.64	0.82	0.98	0.78	0
	**res136**	1	0	0.8	1	0.98	0	0	0	1	0.94
	TYR203	1	0.98	0.54	0.98	0	1	1	0.9	1	1
	ASP204	1	1	0.5	0.08	0	0.84	0.9	1	0.98	0.3
	TYR258	0.1	0.02	0.08	0.22	0	0.06	0.24	0.02	0	0.74
	ARG260	0.02	0.08	0.1	0.04	0	0.08	0.02	0.2	0	0
	GLU138	0	0.04	0.1	0	0	0	0.02	0.94	0.02	0
	GLU281	0	0	0	0.4	0.62	0	0	0	0	0
' + 1'	ARG260	0.4	0.64	0.1	0.36	0.66	0.1	0	0.38	0.06	0
	GLU138	0	0.92	0.04	0.64	0	1	1	0	0.94	0.16
	TYR139	0	0.88	0.72	0.66	0	0.34	0.16	0.4	0.48	0.64
	TYR203	0	0.02	0.02	0	0	0.04	0.04	0	0	0
	ASP204	0	0	0.64	1	1	0.02	0.18	0.96	0.04	0.64
' + 2'	TYR139	0.9	0.02	0.24	0.26	0.96	0.1	0	0.2	0.52	0.32
	ASP204	0.3	0.02	0.96	0.58	1	0.7	0.62	0.82	0	0
	ASN182	0	0.14	0.04	0.38	0.44	0.06	0	0.56	0.18	0
	**res181**	0	0	0	0	0	0	0	0.04	0.02	0
	TYR205	0	0	0	0	0	0	0	0.02	0	0
	GLY179	0	0	0	0	0.06	0	0	0	0	0
' + 3'	**res209**	0.02	0.02	0.12	0	0	0.7	0.94	0	0.28	0
	ASP210	0	0.08	0.76	0	0	0.28	0.06	0.02	0.44	0
	GLN211	0	0	0.1	0	0	0.02	0	0	0	0
	TYR205	0	0	0.06	0	0	0	0	0	0	0
	**res181**	0	0	0	0	0.12	0	0	0	0.26	0
	ASN182	0	0	0	0	0.02	0	0	0	0	0
	**res231**	0	0	0	0	0.04	0	0	0	0.02	0
	GLY207	0	0	0	0	0	0.04	0	0	0	0
Total		9.4	9.4	10.4	10.86	9.26	9.76	9.36	11.54	11.06	8.54

Figure S10. Number of HBs, π- π, cation – π interactions formed during the stable period (40-50 ns) of MD simulation between enzymes and *p*NP-CH3 (**A**) and CH6 (**B**). The figure is colored as a heat map – a higher number is in a more intensive green color, and a smaller number is pale yellow.

Table S3. Average parameters of equilibrated chitinase-substrate complexes.

System	Free energy of binding [kcal/mol]	Number of HBs with protein	Number of HBs with water
WT- *p*NP-CH3	-10.22	9.1	11.2
WT-CH6	-9.57	9.4	22.8
D136V- *p*NP-CH3	-10.27	7.4	10.5
D136V- CH6	-9.19	9.4	24.4
W209A- *p*NP-CH3	-9.73	7.9	10.6
W209A- CH6	-10.11	10.4	21.5
S181W- *p*NP-CH3	-9.39	7.7	11.4
S181W- CH6	-10.05	10.9	21.9
F231W- *p*NP-CH3	-9.76	7.2	10.2
F231W- CH6	-10.14	9.3	20.2
W209A/D136V- *p*NP-CH3	-9.98	9.5	10.6
W209A/D136V- CH6	-9.81	9.4	23.4
S181W/D136V- *p*NP-CH3	-8.48	7.8	12.5
S181W/D136V- CH6	-9.71	11.5	21.8
W209A/F231W- *p*NP-CH3	-9.80	9.0	10.0
W209A/F231W- CH6	-9.60	11.1	25.1
S181W/W209A- *p*NP-CH3	-9.28	4.8	10.3
S181W/W209A- CH6	-8.27	8.5	27.2
S181W/W209A/D136V- *p*NP-CH3	-7.92	9.8	13.1
S181W/W209A/D136V- CH6	-9.61	8.0	24.8

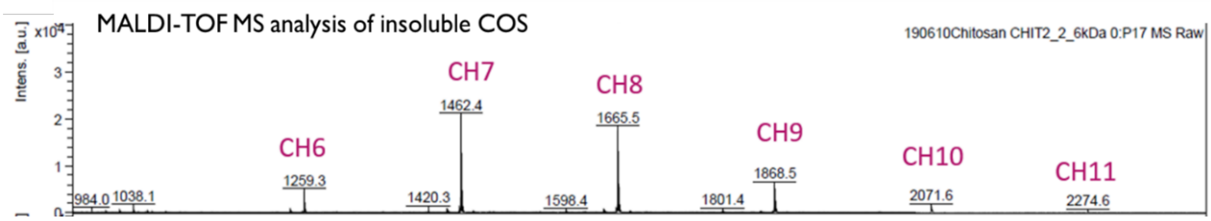

Figure S11. MALDI-TOF MS analysis of a mixture of insoluble chitooligomers (COS) obtained in the reaction catalyzed by the β-*N*-acetylhexosaminidase from *Aspergillus oryzae* (*Ao*Hex) Tyr445Asn starting from CH3. CH6 – CH11 indicates the masses $[M+Na]^+$ of the individual chitooligomers. The electrospray mass spectra were recorded using Q-Tof micro (Waters Milford, MA, USA). The mobile phase consisted of methanol/water (9:1, *v*/*v*), flow rate 100 µL/min, and the samples were injected using a 5-µL loop. The exact masses were measured using LTQ Orbitrap XL hybrid mass spectrometer (Thermo Fisher Scientific, Waltham, MA, USA) equipped with an electrospray ion source. The mass spectra of positively charged ions were internally calibrated using sodium adduct of diisooctyl phthalate ion (*m/z* 413.26623) as a lock mass. The MALDI mass spectra were recorded using UltrafleXtreme (Bruker Daltonics, Bremen, Germany), equipped with 1 kHz smart beam II laser (355 nm). The samples (1µL, 10 mg/mL in water) were mixed with 2,5-dihydroxybenzoic acid (DHB) matrix solution (1 µL, 10 mg/mL in water), deposited on the MALDI plate and left to dry. External mass calibration was done using the Peptide or Protein Calibration Standard supplied by Bruker Daltonics.

Table S4. The *m*/*z* values were monitored by HPLC-MS with CH4, CH5, and MIX-insoluble COS substrate. The values correspond to $[M + H]^+$ of the substrate and deacetylation products. (**A**:GlcNAc; **D**:GlcN).

	Product	$[M + H]^+$		Product	$[M + H]^+$
CH4	A4	831.4	CH6	A6	1237.2
	A2D2	747.4		A3D3	1111.2
	A1D3	705.4		A1D5	1027.2
	Product	$[M + H]^+$		Product	$[M + H]^+$
CH5	A5	1034.98	CH7	A7	1440.2
	A2D3	908.98		A3D4	1272.2
	A1D4	866.98		A1D6	1188.2
